# Somatic cell hemoglobin modulates nitrogen oxide metabolism in the human airway epithelium

**DOI:** 10.1038/s41598-021-94782-5

**Published:** 2021-07-29

**Authors:** Nadzeya Marozkina, Laura Smith, Yi Zhao, Joe Zein, James F. Chmiel, Jeeho Kim, Janna Kiselar, Michael D. Davis, Rebekah S. Cunningham, Scott H. Randell, Benjamin Gaston

**Affiliations:** 1 Herman Wells Center for Pediatric Research, Riley Hospital for Children, Indiana University School of Medicine, 1044 W. Walnut Street, R4-474, Indianapolis, IN 46202 USA; 2 Case Western Reserve University, Cleveland, OH USA; 3 University of North Carolina School of Medicine, Chapel Hill, NC USA; 4 Respiratory Institute, Cleveland Clinic, Cleveland, OH USA

**Keywords:** Biochemistry, Biological techniques, Biotechnology, Structural biology, Diseases, Molecular medicine

## Abstract

Endothelial hemoglobin (Hb)α regulates endothelial nitric oxide synthase (eNOS) biochemistry. We hypothesized that Hb could also be expressed and biochemically active in the ciliated human airway epithelium. Primary human airway epithelial cells, cultured at air–liquid interface (ALI), were obtained by clinical airway brushings or from explanted lungs. Human airway Hb mRNA data were from publically available databases; or from RT-PCR. Hb proteins were identified by immunoprecipitation, immunoblot, immunohistochemistry, immunofluorescence and liquid chromatography- mass spectrometry. Viral vectors were used to alter Hbβ expression. Heme and nitrogen oxides were measured colorimetrically. Hb mRNA was expressed in human ciliated epithelial cells. Heme proteins (Hbα, β, and δ) were detected in ALI cultures by several methods. Higher levels of airway epithelial Hbβ gene expression were associated with lower FEV_1_ in asthma. Both Hbβ knockdown and overexpression affected cell morphology. Hbβ and eNOS were apically colocalized. Binding heme with CO decreased extracellular accumulation of nitrogen oxides. Human airway epithelial cells express Hb. Higher levels of Hbβ gene expression were associated with airflow obstruction. Hbβ and eNOS were colocalized in ciliated cells, and heme affected oxidation of the NOS product. Epithelial Hb expression may be relevant to human airways diseases.

## Introduction

In systemic vascular endothelial cells, hemoglobin (Hb)α and endothelial nitric oxide synthase (eNOS) are colocalized adjacent to the myoendothelial junction. Hemoglobin α modulates eNOS activity by regulating the redox state of the NOS product: eNOS is activated in the presence of Hbα-Fe^2+^ produces primarily inert NO_3_^−^, whereas eNOS in the presence of Hbα-Fe^3+^ produces active NO and S-nitrosothiols (SNOs)^1^. In the airway epithelium, we and others have shown that eNOS is expressed at the base of cilia^2,3^, where it has a role in regulating epithelial protein expression and hydration, i.e. water and ion transport to the airway surface layer^3^. The bioactivities of nitrogen oxides produced by eNOS and by inducible NOS (iNOS) in the airway are somewhat controversial, but products of both enzymes are generally believed to augment host defense and airway smooth muscle relaxation^4,5^. We hypothesized that nitrogen oxide biochemistry could be modulated in the airway epithelium by somatic cell Hb, as it is in the endothelium. Hemoglobin has been identified in the A549 airway cancer cell line but has not previously been studied in primary human ciliated airway epithelium. We were particularly interested in the possibility that Hb could be expressed in ciliated epithelium because it could modify chemistry downstream of NOS activity, as it does in the vasculature, nitrogen oxide signaling has been difficult to understand in a number of airways diseases including asthma^6^, cystic fibrosis (CF)^3^, sickle cell anemia^7^, and primary ciliary dyskinesia (PCD)^8^.

## Materials and methods

### Materials

Materials were purchased from Sigma-Aldrich (St. Louis, MO) unless otherwise noted.

### Cell culture

Primary normal human airway epithelial cells were grown from human bronchial brushings, nasal brushings or from explanted lung tissue as previously described^9^. Specimens were isolated by protease dissociation from nasal brushings or explanted lungs as previously described^9^ under protocols approved as described below (Study Permissions). Subjects included two healthy, non-smoking control subjects and two subjects with Primary Ciliary Dyskinesia (PCD; genotypes: compound heterozygous for DNAH5 and CCDC39). In some cases, explanted bronchial cells were purchased commercially (Lifeline Cell Technologies, Massachusetts, USA). Cells were grown at air–liquid interface (ALI) until fully pseudostratified and ciliated. In some experiments, cells were treated in a sealed chamber with 1% CO (to bind heme) for 10 min, then returned to the incubator for 5 h. Human airway smooth muscle cells cultures were purchased from American Type Tissue Culture (ATCC PCS-130-010) and grown as previously described^10^.

### In silico RNA-seq data acquisition and analysis

Gene expression data were acquired from the GEO database^11^ (https://www.ncbi.nlm.nih.gov/geo/query/acc.cgi?acc=GSE97036) series GSE97036 of 48 samples collected from six healthy donors. The expression data of hemoglobin β (HBβ) and α (HBα) were then extracted from the datasets. The boxplots were created using R version 4.0.2. The comparison of HBβ expression level between groups was conducted using a negative binomial regression. The p-values were corrected for pair-wise comparison following the Holm-Bonferroni method. Single-cell RNA expression data from human airway cells grown at ALI were acquired from the publicly available Klein lab database^12^ (https://kleintools.hms.harvard.edu/tools/springViewer_1_6_dev.html?datasets/reference_HBECs/reference_HBECs) using SPRING plots available in the database. SPRING is a tool for uncovering high-dimensional structure in single-cell gene expression data. SPRING takes a (gene X cell) table of expression measurements and outputs a k-nearest-neighbor graph rendered using a force directed layout. Users can overlay prior information, including gene expression values, gene-set scores, cell cluster labels and sample IDs. Users can also upload custom coordinates that have been generated using an outside program such as tSNE.

### RT-PCR

RNA was isolated from airway epithelial cells using Qiagen kit (Qiagen, # 69504). The Taq-Man Gene expression master mix (AB Applied Biosystems, #4369016) was used according to the supplier’s instructions with the Hbβ primers 5′-GTTTAGCCAGGGACCGTTTCAG and 5′-AATTCTGGCTTATCGGAGGCAAG. Human ACTB (Beta Actin) (VIC®/MGB probe, primer limited, Fisher # 4326315E) was used as endogenous control. The Hbβ mRNA was expressed as ratio to actin mRNA.

### Hemoglobin over-expression and knockdown in primary human airway epithelial cell cultures

A retroviral vector expressing Hbβ (pQCXIN w/ CMV promotor tagged with Myc & Flag + Puromycin resistance gene) or empty vector control (pQCXIP empty vector), and lentiviral vectors containing four separate short hairpin RNA (shRNA) sequences, and their respective control vectors were obtained through the Vector Core at the University of North Carolina at Chapel Hill. Normal human bronchial epithelial cells grown on collagen type I/III coated 60 mm dishes at 50% confluence, were incubated with polybrene/virus mixture (1:500 ratio) for 3 h at 37 °C with gentle agitation every 30 min. Cells were then washed with PBS and cultured for 48 h before starting selection with 1ug/ml puromycin in culture media. After selection and once the cells reached 90% confluence, they were passaged onto type IV collagen coated 12 mm porous supports (Millipore, Millicell CM) in ALI media with puromycin. Cells were grown at an ALI under continued puromycin selection until differentiated with mucoid secretions and with visible beating ciliated cells, at which point they were lysed or fixed to assess the level of Hbβ expression and morphology.

### Immunoprecipitation and immunoblot

NHBE cells grown at ALI were lysed with RIPA buffer and protease inhibitors. Cell lysates underwent immunoprecipitation (IP) for 2 h with primary anti eNOS MAB (BD Transduction, #610296, 1:500) or with primary anti Hbβ monoclonal antibodies (MAB; AbCam, #Ab100952, 1: 500), washed 3 × in PBS, extracted from beads with Laemmli buffer, put in 2 × loading buffer, and run on a 4–15% SDS-page gel,. The membrane was incubated overnight with primary antibodies. We used primary anti Hbβ MAB (AbCam, #Ab100952, 1:1000) with secondary (Santa Cruz, SC-2061, 1:3000); primary anti eNOS MAB (BD Transduction, #610296, 1:1000) with secondary (Santa Cruz, SC-2060, 1: 3000). Blots were visualized using the Biorad Chemidoc system.

### Liquid chromatography–mass spectrometry proteomics

ALI cell lysates were immunoprecipitated with Hbβ antibody as above (AbCam, #ab100952) for 2 h at four degrees with rotation. After this, they were incubated at four degrees overnight with washed protein G agarose beads (Invitrogen, 15920-010) and washed several times with PBS. After boiling in Laemmli buffer, the samples were run on a 4–15% SDS-page gel with 50 ug total protein and lysed RBCs as controls. The gels were fixed and stained with Coomassie. Excised gel bands were in-gel digested using 150 ng of trypsin per each band as described by M. Mann group^13^. LC–MS analysis of all digested protein bands was carried out on the Orbitrap Elite mass spectrometer (Thermo Electron, San Jose, CA) interfaced with a Waters nanoAcquity UPLC system (Waters, Taunton, MA). Proteolytic peptides from each gel band were desalted on a trap column (180 μm × 20 mm packed with C18 Symmetry, 5 μm, 100 Å (Waters, Taunton, MA)) and subsequently eluted on a reverse phase column (75 μm × 250 mm nano column, packed with C18 BEH130, 1.7 μm, 130 Å (Waters, Taunton, MA)) using a gradient of 2–42% mobile phase B (0.1% formic acid and acetonitrile (ACN)) versus mobile phase A (100% water/0.1% formic acid) over a period of 60 min at 40 °C with a flow rate of 300 nl/min. Proteolytic peptides eluting from the column were introduced into the nano-electrospray source at a capillary voltage of 2.5 kV. A full scan for MS analysis was recorded for eluted peptides at the m/z range of 350–1800 and resolution R of 120,000 followed by MS/MS of the twenty most intense peptide ions scanned in the ion trap mass analyzer. The resulting MS/MS data were searched against IPI human proteins database using Mascot engine to identify proteins from each gel band with the mass accuracy of 10 ppm and 0.8 Daltons for MS and MS/MS scans respectively, and with allowed variable modifications including carbamidomethylation for Cys and oxidative modification for Met.

### Chemical measurements

In both human airway epithelial cells and human airway smooth muscle cells, both medium and intracellular nitrate (NO_3_^−^) was measured reductively, and nitrite (NO_2_^−^) was measured on samples by Griess reagent, as reported previously^14^. Cell lysate measurements were performed with or without pre-incubation with and without the NOS inhibitor, nitro-L-arginine methyl ester (L-NAME)^20^. Cell viability was determined by LDH assay in accordance with the manufacturer’s instructions (Cayman Chemical, cat#601170). The Drabkin assay was performed as previously described^15^ on lyophilized whole cell lysate. Cyclic GMP assays were performed colorimetrically using the enzyme-linked immunosorbent assay (ELISA) kit from R&D systems, including the standards provided (lower limit of detection, 2 pM).

### Immunohistochemistry (IHC)

Paraffin-embedded tissue sections of human lung biopsy sections (local samples obtained during research bronchoscopies^16^) on slides were deparaffinized in 2 changes of xylene, 5 min each; slides were transferred to 100% ethanol for 2 changes, 3 min each, and then transferred once through 95%, 70%, and 50% ethanol respectively for 3 min each. Endogenous peroxidase activity was blocked by incubating sections in 3% H_2_O_2_ solution in methanol at room temperature for 10 min to block endogenous peroxidase activity. Slides were rinsed in 300 ml of PBS for 2 changes, 5 min each; blocked in 10% fetal bovine serum in PBS) for 1 h; incubated with eNOS mouse MAB (1:200) (BD Transduction Laboratories # 610296) using secondary goat anti-mouse AB (1: 10,000; BD Pharmingen # 554002; 30 min, RT) and ABC reagent (Vector Labs) for 30 min, (RT), using manufacturers protocol. Cross-sectional images from paraffin-embedded ALI cultures under- or over-expressing Hbβ were stained with AB-PAS. Number of ciliated cells was counted by three investigators blinded to sample identity.

### Immunofluorescence

Primary NHE cells were fixed in 4% paraformaldehyde for 30 min, permeabilized in 0.05% Triton-X for 5 min, rinsed, and incubated overnight with primary antibodies to Hbβ (MAB; AbCam, #Ab100952, 1:500) and eNOS MAB (BD Transduction, #610296, 1:200). After rinsing, they were incubated with secondary antibodies (Alexa Fluor 568, 1:100 dilution; Alexa Fluor 488, 1:500 dilution; Invitrogen, Carlsbad, CA) and visualized with confocal microscopy, Leica. In separate studies at UNC, whole mount immunostaining of ALI cultures and confocal imaging was performed using Sigma, MAB, #7B12HBβ (5 μg/ml) for Hbβ, with co-staining for αtubulin, filamentous actin and nuclei as previously described (PMID: 29481290). Empty vector treated cell cultures and isotype IgG (5 μg/ml) were used as negative controls.

### Severe asthma study population

Gene expression (mRNA) from bronchoscopic brush biopsies as well clinical data were obtained and characterized from subjects in the National Institute of Health-National Heart, Lung, and Blood Institute (NIH-NHLBI) Severe Asthma Research Program (SARP) from 2009 to 2011^17,18^. Clinical characteristics of SARP I&II participants on whom gene expression data from bronchial epithelial cell are publicly available in NCBI's Gene Expression Omnibus^19^ (https://www.ncbi.nlm.nih.gov/geo/query/acc.cgi?acc=GSE63142) and are provided in Supplementary Table 1. (Supplemental Material available at URL: http://dx.doi.org/10.17632/d86hc73sk8.1). Details on sample preparation and microarray experiments have been previously described^17^.

### Study permissions

The clinical study to obtain nasal brush biopsies was approved by the University of North Carolina at Chapel Hill Biomedical Institutional Review Board (#03-1396) and by the University Hospitals Cleveland Medical Center (IRB # 10-04-14). The SARP protocols, including brush biopsies for mRNA analysis and endobronchial forceps biopsies, were approved at each individual SARP I and II centers as described previously^20^. Written informed consent was obtained from all participants.

All methods were carried out in accordance with relevant guidelines and regulations. (Declaration of Helsinki).

### Statistical analysis

For the in vitro data, t-tests or nonparametric rank sum tests were used for two-sample comparison of continuous variables: a Fisher exact test was used for two-sample comparison of categorical variables and analysis of variance was used for categorical variables with more than two categories. *p* ≥ 0.05 was considered statistically significant.

For the biochemical assays, we used the Tukey’s method. We first fitted a robust ANOVA model with three-way interactions (group, cytomix, and CO) and then applied the Tukey’s test which corrects for multiple comparison. Here we used a robust ANOVA model as the data variation is not homogenous.

Publicly accessible databases (Klein lab database^12^ and GEO database^11^) were analyzed by their curators as previously published. For the ex vivo SARP data, correlation analyses of expression levels of hemoglobin genes Hbα1, Hbα2, Hbβ and Hbδ, and pre-bronchodilator percent of predicted forced expiratory volume in 1 s (FEV_1_PP) in cells from human bronchial epithelial biopsy of patients with asthma (n = 128) and control (n = 27) enrolled in SARP. Multiple linear regression models were used to evaluate the association between gene expression levels of Hb genes and pre-bronchodilator (Pre-BD) FEV_1_PP, adjusting for age, sex, body mass index (BMI) and race. *P* values < 0.05 were considered statistically significant. Adjustment to multiple testing was done using Bonferroni correction. Analyses were performed using R statistical software (R Foundation for Statistical Computing, Vienna, Austria).

## Results

### Expression of hemoglobin genes and proteins in human airway pseudostratified epithelium in vitro

**Figure 1:**
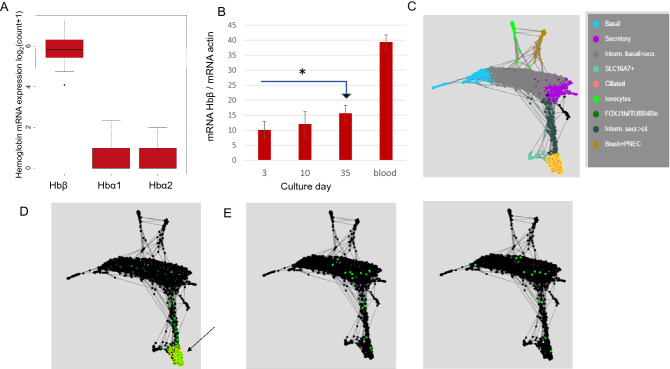
Hemoglobin RNA is expressed in human airway epithelium and increases during maturation to fully ciliated epithelium. (**A**) Box and whisker plots show hemoglobin β (Hbβ), hemoglobin α1 (Hbα1) and hemoglobin α2 (Hbα2) gene expression data (log2 transformed) of 48 samples collected from six healthy donors (GEO database^11^: https://www.ncbi.nlm.nih.gov/geo/query/acc.cgi?acc=GSE97036). *p < 0.001. Box and whisker plots were created using R Software: R Core Team, 2021. R: A language and environment for statistical computing. R Foundation for Statistical Computing, Vienna, Austria. URL https://www.R-project.org/. (**B**) Reverse transcription polymerase chain reaction (RT-PCR) analysis of Hbβ expression normalized to actin levels in primary normal human airway epithelial cell ALI cultures at days 3, 10, and 35; mRNA Hbβ obtained from human blood was a control (n = 6, *p ≤ 0.0047). (**C**) SPRING plot from publicly available single cell gene cluster analysis of human bronchial epithelial cell ALI cultures^12^. Each dot is a single cell. Blue-basal cells, purple-secretory, green-ionocytes, brown-brush + neuroendocrine cells, yellow-orange-ciliated cells. (**D**) Hbβ gene expression in primary human bronchial epithelial cells^12^. Each dot is a single cell. The distribution of Hbβ in HBEC: Hbβ (green) is predominantly localized in ciliated cells; that is, it has the same distribution as the yellow-orange cells in (**C**). Arrow points to Hbβ in ciliated epithelium. The SPRING plot is queried for Hbβ (*HBB*) mRNA. (**E**) Hemoglobin α1 and α2 gene expression in primary human bronchial epithelial cells. There is less Hbα1 and Hbα2 (green) HBEC: Hbα1 (left, green) and Hbα2 (right, green), and they are not localized to ciliated cells. The SPRING plot is queried for Hbα1 and Hbα2 (*HBA1* and *HBA2*) mRNA.

To begin with, we have identified Hb gene expression in human airway epithelial cells in publicly accessible databases and confirmed it with RT-PCR. Hemoglobin β (Hbβ) RNA, hemoglobin α1 (Hbα1) RNA and hemoglobin α2 (Hbα2) RNA expression, shown as box-and-whisker plots, were expressed in human airway epithelium collected in 48 samples of the healthy donors and obtained from the GEO database, Series GSE97036 (https://www.ncbi.nlm.nih.gov/geo/query/acc.cgi?acc=GSE97036). See also ref 11. Hemoglobin β was the predominant form (*p* < 0.001) (Fig. 1A). We confirmed Hbβ expression in human airway pseudostratified cultures at ALI using RT-PCR: Hbβ mRNA expression increased with epithelial maturation (n = 6, *p* ≤ 0.0047), at day three compared to day 35, when airway epithelial cells reach full maturation and become ciliated^21^ (Fig. 1B). Note that the process of airway epithelial differentiation in the ALI culture model is visible by phase contrast microscopy^9,12^. It involves growth from sub-confluence to a confluent culture with mucoid secretions and visibly beating multiciliated cells over a period of > 21 days. In these cells, FOXJ1 increases dramatically by day 35 in the primary cells^21^, correlating with visible multiciliated cells in the primary cell cultures but not in the cell line. Hbβ is expressed at the 35-day time point in the primary cells (Fig. 1B). Additionally, using SPRING plot analysis of a public database for single cell human airway epithelial culture gene expression (Kleintools software; https://kleintools.hms.harvard.edu/tools/springViewer_1_6_dev.html?datasets/reference_HBECs/reference_HBECs)^12^ Hbβ expression was identified primarily in ciliated human airway epithelial cells (HBEC) (Fig. 1C, D). In this figure, single cell gene expression data from human airway epithelial cells grown to pseudostratified cultures at ALI are clustered by gene expression profiles into cell types. Ciliated cells are shown in yellow-orange (Fig. 1C), where Hbβ is also strongly expressed (Fig. 1D). Hemoglobin α1 mRNA and hemoglobin α2 mRNA were also expressed, but much less than Hbβ, and very little were present in ciliated cells (Fig. 1E, right and left).

**Figure 2:**
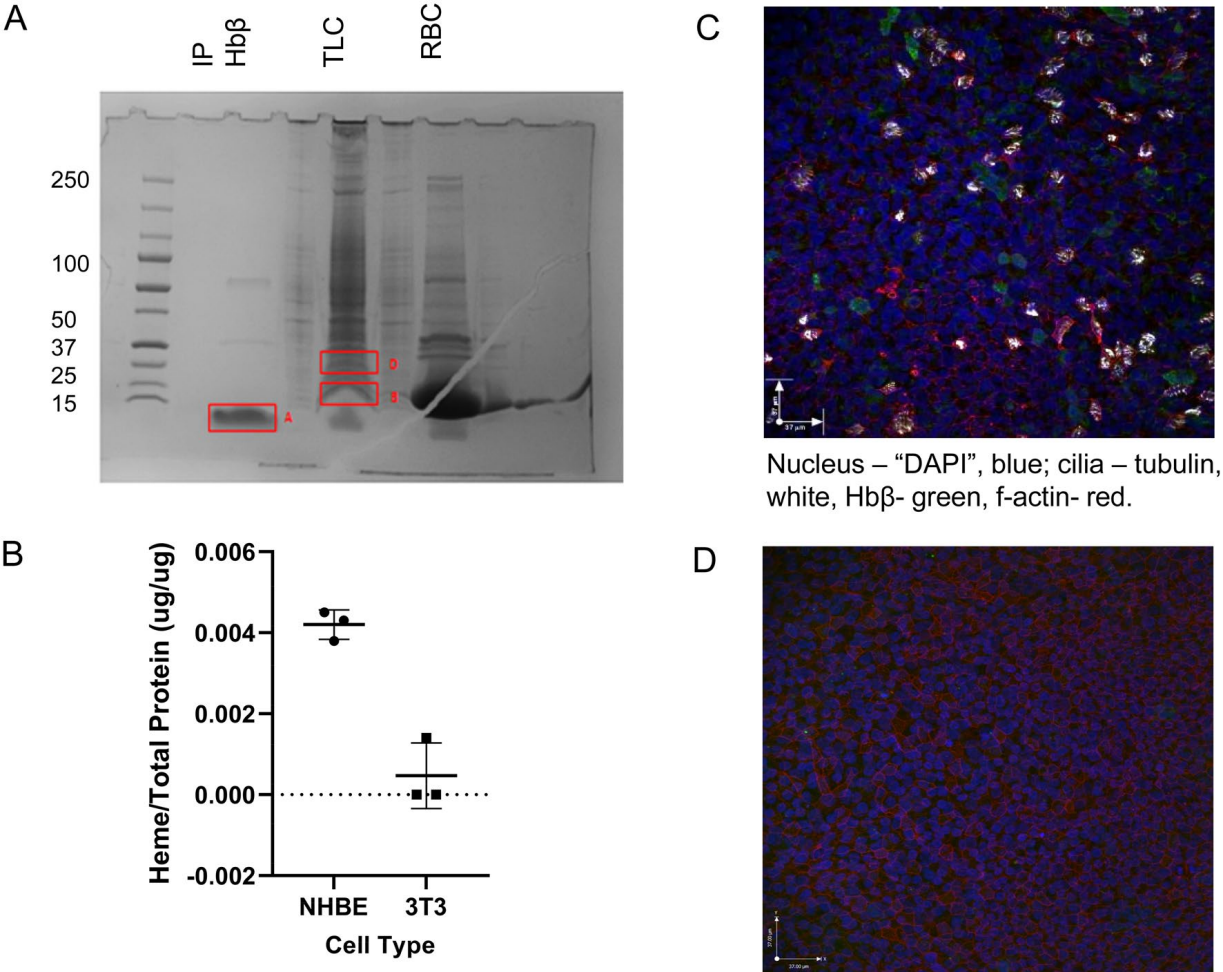
Heme proteins in human airway epithelial cell cultures were detected by immunoblot, immunofluorescence and by LC–MS proteomics. (**A**) Hbβ was immunoprecipitated (IP Hbβ) and run with total lysate from cells (TLC) and whole erythrocyte lysate (RBC) on an SDS gel, followed by Coomassie blue staining. Three bands were excised from the immunoprecipitated Hbβ (IP Hbβ) band and TLC for LC–MS. LC–MS analysis of the samples reveals: Band A: Hbβ, Hbδ, Hbα, Protein S100-A6. Bands B and D: Hbβ, Hbδ, Hbα and other proteins. (**B**) Airway cell proteins have bound heme**.** Drabkin reagent (Sigma, # D5941) was used to determine average amount of heme/total protein^15^. Three normal primary human airway epithelial cell samples, consisting of 10–12 filter each scraped in RIPA buffer, were lyophilized and reconstituted in Drabkin reagent. Heme was measured with absorbance (λ = 540 nm) by spectrophotometer. The fibroblast NIH 3T3 cells were used as a control. (**C**) Immunofluorescent staining of primary normal human airway epithelial cells grown for 35 days at ALI, then fixed, permeabilized and labeled as described in the methods, confirmed expression Hbβ in HBE cells (top). (Nucleus—DAPI, blue; cilia—white, HBβ-green, f-actin red). (**D**) Isotype control IgG (for the anti-Hb Ab) in cells grown identically to those in 2D served as a negative control.

To determine whether the gene expression data were reflected in protein expression, we performed a number of experiments. In the airway epithelial immunoprecipitate, most of the Hb β is monomeric, but there are faint trimer and tetramer bands as well (Fig. 2A). In the erythrocyte lysate, the majority is dimeric*.* To analyze this further, we performed IP and Coomassie staining on the proteins and cut out the Hb band for LC–MS proteomic analysis. Hb α, β, and δ proteins were expressed in human airway pseudostratified epithelial cultures (Fig. 2A). Note that heme was also present in human airway epithelial cells at ALI: concentrations were substantially higher in NHE cells (heme amount 0.0042 ± 0.00035 μg/μg protein) than in NIH 3 T fibroblasts (0.00047 ± 0.00083 ug/ug protein), (Fig. 2B). Note that heme could have been incorporated into non Hb proteins in airway epithelial cells. Finally, Hbβ was also expressed in ciliated cells detected by immunohistochemistry in NHE cells (Fig. 2C); Fig. 2D is a negative control for IHC Hbβ.

### Epithelial Hb gene expression in human asthma

**Figure 3:**
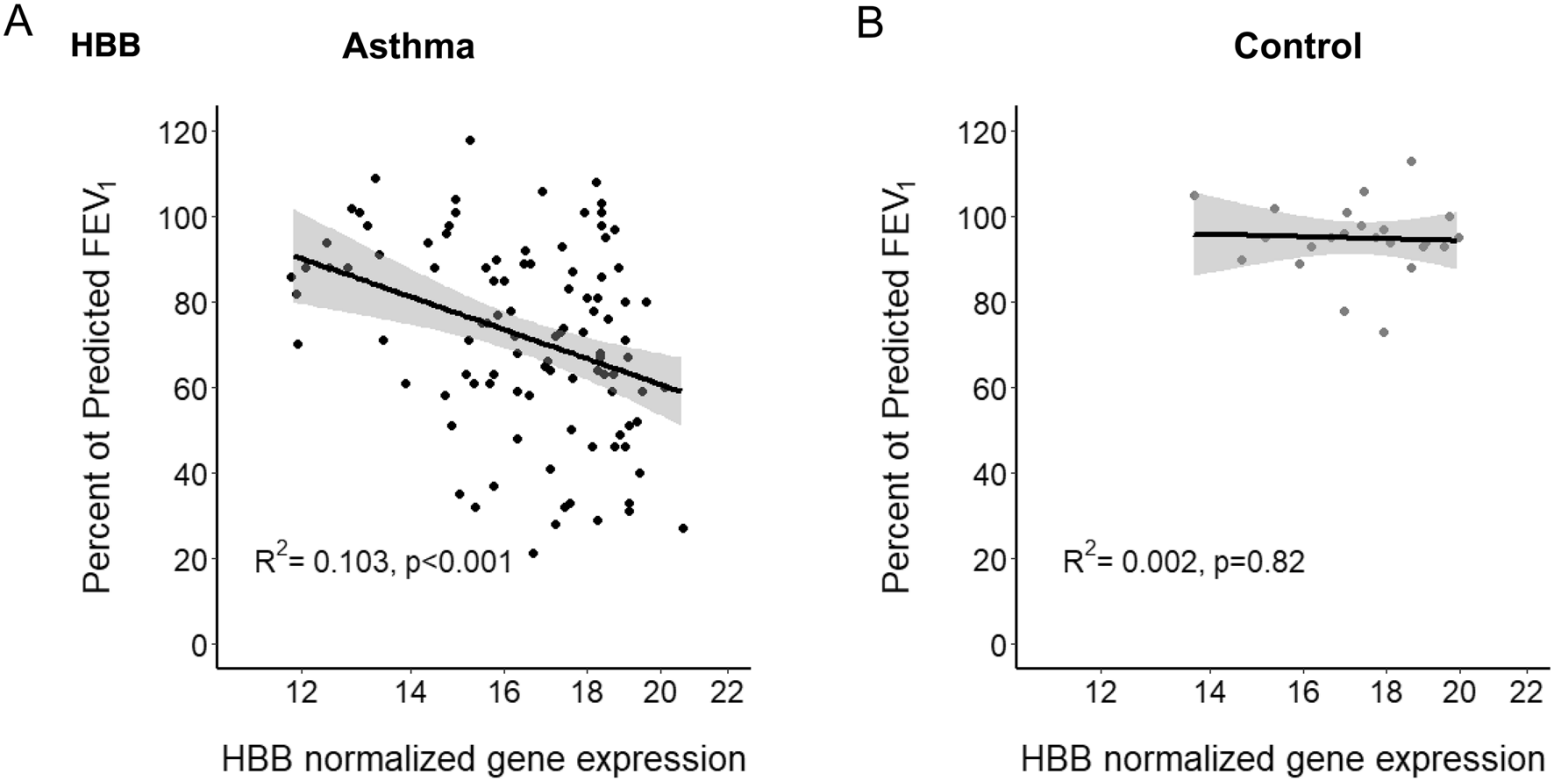
The expression of hemoglobin B gene (HHBB)in bronchial epithelial cells. In samples collected during Severe Asthma Research Program (SARP) bronchoscopies and now available in a publicly accessible database^18^, HBB gene expression strongly correlated with each individual subject’s matched pre-bronchodilator FEV_1_ percent predicted in patients with asthma (**A**); but this was not the case for controls (**B**) (asthma: R^2^ = 0.103, *p* < 0.001, control R^2^ 0.002, *p* = NS).

In bronchial epithelial cells collected by bronchoscopic brush biopsy, the expression of hemoglobin genes (HBA1, HBA2, HBB and HBD) was strongly correlated with Pre-bronchodilator FEV_1_ PP in patients with asthma enrolled in SARP I&II but not in controls (Fig. 3A, B; Supplementary Fig. 1). This association continued to be significant even after adjustment for multiple testing and for age, sex, race, and BMI. (Supplementary Table 1, 2). (Supplemental Material available at URL: http://dx.doi.org/10.17632/d86hc73sk8.1).

### Potential roles for endogenously expressed Hb in airway epithelial cells

#### Morphology and development

**Figure 4:**
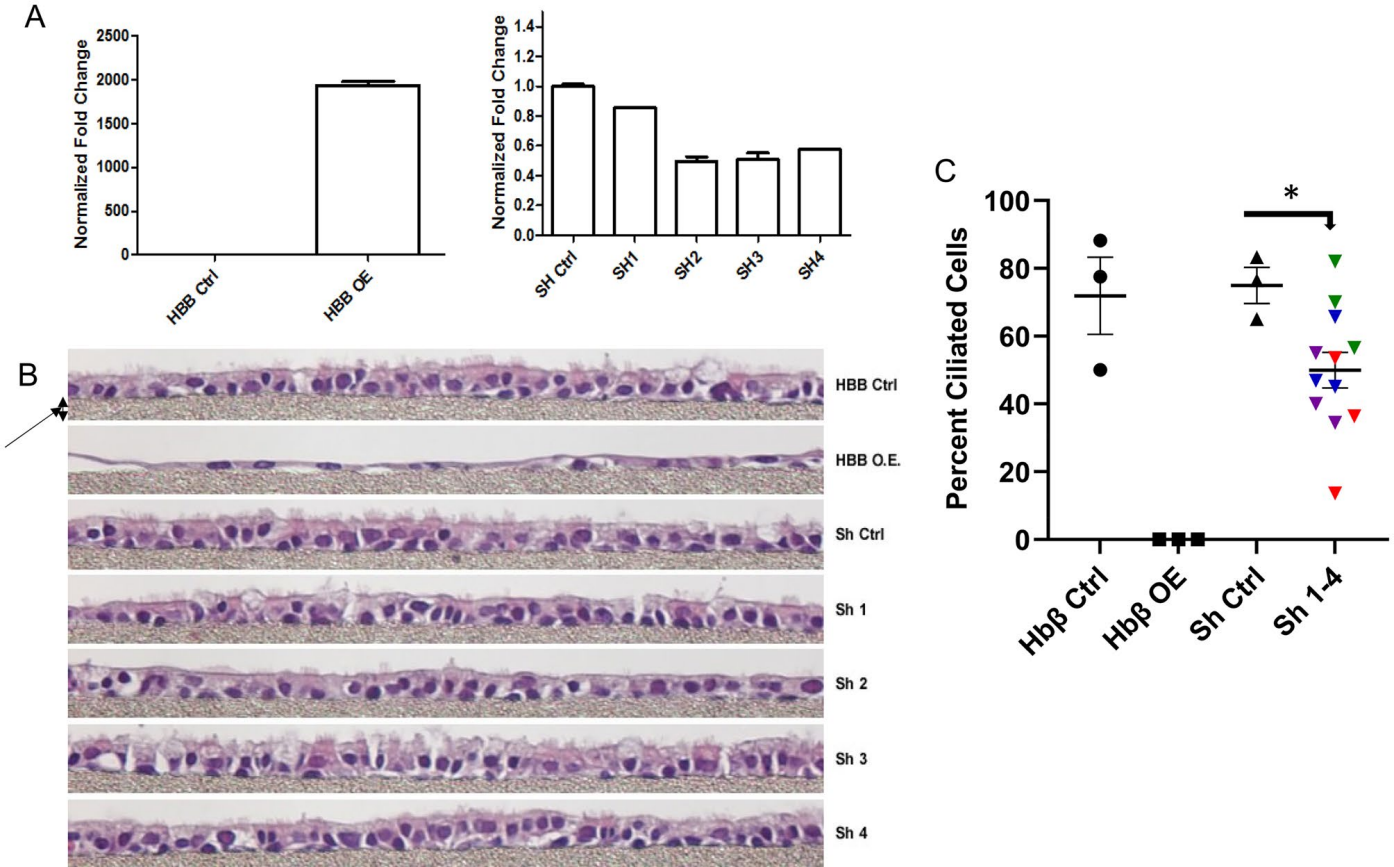
Both hemoglobin β overexpression and hemoglobin β knockdown decrease ALI ciliation. (**A**) RT-PCR analysis of Hbβ mRNA expression in Day 33 ALI primary human bronchial epithelial cells at ALI, transduced with empty vector (Hbβ Ctrl), Hemoglobin β over-expressed vector (Hbβ O.E.), Scrambled Ctrl (SH Ctrl), and Hemoglobin β knockdown shRNA ‘s 1-4 (SH1-4). (**B**) Representative AB-PAS staining of paraffin embedded sections of Day 33 ALI cultures from the Hbβ under- and over-expressing, and control, cells as described in (**A**). 20 × magnification. Arrow shows the membrane thickness that is equal 20 μm. These images were read by three blinded observers each, counting the number of ciliated cells as a fraction of cells on the apex of the culture (see **C**). (**C**) Percentage of ciliated apical cells were quantitated blindly by three independent investigators. Cilia were absent from the Hbβ over-expressing cells. In the Hbβ knockdown cells, ciliary expression was decreased relative to wt and scrambled shRNA expressing cells, shown in aggregate (Purple is Sh1, Red is Sh2, Green is Sh3, and Blue is Sh4), (*p = 0.041).

We studied the effect of both Hbβ overexpression and Hbβ knockdown (Fig. 4A) on airway epithelial cell morphology. As noted above, we used 33 - 35 day ALI cultures, when cells had achieved full maturation^9^. These cells did not preserve or passage well. Overexpression dramatically decreased epithelial differentiation and ciliation (Fig. 4B, C). Overall, knockdown also decreased airway epithelial ciliation relative to scrambled siRNA control, though we never achieved more than 50% knockdown, *p* = 0.041 (Fig. 4B, C). Coupled with evidence that Hbβ gene expression increases with epithelial maturation (Fig. 1), these data suggest that Hbβ could have a role in airway epithelial maturation and ciliation.

#### Nitrogen oxide chemistry

**Figure 5:**
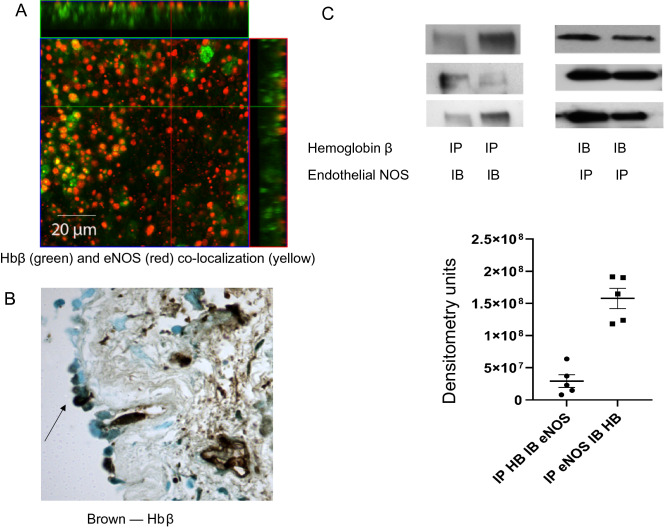
Hemoglobin β is colocalized with endothelial nitric oxide synthase (eNOS) in normal human airway epithelial cells. (**A**) Immunofluorescent staining of Hbβ (green) and eNOS (red) are co-localized (yellow) in primary airway cells NHE grown on ALI, permeabilized, fixed and labeled as described in the methods. Studies were performed by confocal microscopy, Leica, 60X. (**B**) Immunohistochemical staining of Hbβ in an endobronchial biopsy from a healthy subject in the SARP. Normal lung biopsy revealed presence of Hbβ at the apex of airway epithelial cells (arrow) and some in inflammatory cells, × 40 magnification. (**C**) Colocalization of eNOS and Hbβ in normal human airway epithelial cell cultures. Cell cultures at ALIs were lysed in RIPA buffer and immunoprecipitated (IP) for Hbβ followed by immunoblot (IB) for and eNOS (left panel); in separate cells, proteins were IP’d for eNOS followed by IB for Hbβ (right panel). Immunoprecipation and IB were performed as described in the methods, total. N = 5.

In endothelial cells, Hb regulates NOS activity^1^. We studied whether it could have a similar role in the airway epithelium. It is known that eNOS is expressed apically, at the base of cilia, in ciliated epithelium^2,3^. We therefore sought to determine whether Hb was colocalized with eNOS. First, we determined that Hbβ was expressed apically by IF and IHC (Fig. 5A, B). Next, we determined that Hbβ was colocalized with eNOS in NHE ALI cultures, both by immunofluorescence and co-immunoprecipitation (Fig. 5A, C).

**Figure 6:**
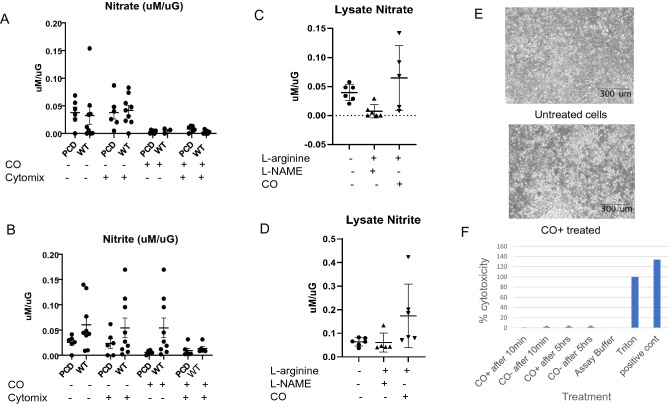
Carbon monoxide (CO) prevents NO oxidation in human primary airway epithelial cell culture medium but does not affect cell viability or NOS activity. (**A**) Nitrate accumulation in the extracellular medium did not differ between subjects (PCD vs wt, *p* = NS for each pair-wise comparison). Surprisingly, cytomix (10 ng/uL each of TNFα, IFNγ and LPS for 4 h) did not increase NO_3_^−^ in these primary pseudostratified ALI cultures, in contrast to previous reports from our group and others^20,21^ regarding submerged cell lines. This argues against iNOS being the principal source of nitrogen oxides in this system. However, CO (1 ppm; 10 min) completely inhibited NO_3_^−^ production overall (n = 21 positive vs n = 30 negative, *p* < 0.001). For the pair-wise comparisons, the inhibition effect of CO was significant in PCD without cytomix (*p* = 0.001, Tukey *p* = 0.002) and WT with cytomix (*p* < 0.001, Tukey *p* < 0.001) after Tukey’s multiple correction; was significant in PCD with cytomix before Tukey’s correction (*p* = 0.017, Tukey *p* = 0.067), and insignificant in WT without cytomix (*p* = 0.355, Tukey *p* = 0.827). (**B**) Carbon monoxide (as above) also inhibited NO_2_^−^ accumulation in the medium (4 h, as above; *p* < 0.001). For the pair-wise comparison, CO significantly inhibited NO_2_^−^ production in PCD without cytomix (*p* = 0.009, Tukey *p* = 0.035) and WT without cytomix (*p* < 0.001, Tukey *p* < 0.001) after Tukey’s correction; and significantly inhibited NO_2_^−^ in WT with cytomix before Tukey’s correction (*p* = 0.035, Tukey *p* = 0.131), while no significant difference was observed in PCD with cytomix (*p* = 0.071, Tukey *p* = 0.255).” (**C, D**) Though CO can also bind heme in NOS^22^ the effect of NOS substrate, l-arginine (1 mM), to increase intracellular nitrogen oxides NO_3_^−^ (**C**) and NO_2_^−^ (**D**) was completely ablated by excess NOS inhibitor nitro-L-arginine methyl ester (L-NAME; 10 mM), but not by CO (NO_3_^−^: L-NAME vs CO; n = 5; *p* = 0.019); (NO_2_^–2^; L-NAME vs CO; n = 5; *p* = 0.0015). (**E, F**) Note that 10 min of CO pretreatment had no effect on the viability of these cells in culture, either by phase contrast imaging (**E**) or by Triton blue exclusion (**F**).

To study the effect of heme on nitrogen oxide metabolism^1^, we exploited the fact that Hb Fe^2+^ avidly binds CO, preventing its oxidation to Fe^3+^ and formation of NO oxidation products in the presence of oxygen. Thus, CO should prevent NOS-derived NO oxidation. We began by measuring the effect of CO on nitrogen oxide accumulation in the cell culture medium. In Fig. 6A, we show that NO_3_^−^ accumulation in the extracellular medium did not differ between subjects (PCD vs wt, *p* = NS for each pair-wise comparison). Surprisingly, basolateral cytomix (10 ng/uL each of TNFα, IFNγ and LPS for 4 h) did not increase NO_3_^−^ in these primary pseudostratified ALI cultures, in contrast to previous reports from our group and others^20,21^ regarding submerged cell lines. This argues against iNOS being the principal source of nitrogen oxides in this system. However, CO (1 ppm; 10 min) completely inhibited NO_3_^−^ production overall (n = 21 positive vs n = 30 negative, *p* < 0.001). For the pair-wise comparisons, the inhibition effect of CO is significant in PCD without cytomix (*p* = 0.001, Tukey *p* = 0.002) and WT with cytomix (*p* < 0.001, Tukey *p* < 0.001) after Tukey’s multiple correction; was significant in PCD with cytomix before Tukey’s correction (*p* = 0.017, Tukey *p* = 0.067), and insignificant in WT without cytomix (*p* = 0.355, Tukey *p* = 0.827). Carbon monoxide (as above) also inhibited NO_2_^−^ accumulation (Fig. 6B) in the medium (4 h, as above; *p* < 0.001). For the pair-wise comparison, CO significantly inhibited NO_2_^−^ production in PCD without cytomix (*p* = 0.009, Tukey *p* = 0.035) and WT without cytomix (*p* < 0.001, Tukey *p* < 0.001) after Tukey’s correction and significantly inhibited NO_2_^−^ in WT with cytomix before Tukey’s correction (*p* = 0.035, Tukey *p* = 0.131), while no significance was observed in PCD with cytomix (*p* = 0.071, Tukey *p* = 0.255).

Though CO can also bind heme in NOS the effect of excess NOS substrate, the effect of l-arginine treatment (1 mM) to increase intracellular nitrogen oxides (NO_3_^−^ and NO_2_^−^)^22,23^ was completely ablated by excess NOS inhibitor nitro-L-arginine methyl ester (L-NAME; 10 mM), but not by CO (Fig. 6C, D). Note that 10 min of CO pretreatment had no effect on the viability of these cells in culture (Fig. 6E, F). Note also that we performed these studies in airway epithelial cell cultures from patients with PCD to determine whether decreased NO production in the PCD airway^8^ could be associated with increased Hb; the results were similar, though extracellular NO_2_^−^ were somewhat lower in PCD, particularly in the presence of CO (Fig. 6B). Finally, note that upregulation of iNOS by cytomix^22,23^ did not affect CO-inhibitable NO_3_^−^ formation (Fig. 6A, B), suggesting that the source of the NO was subciliary eNOS, consistent with previous publications^3^ and with the colocalization data shown above (Fig. 5).

## Discussion

Hemoglobins have been identified in a variety of mammalian somatic cells^24–32^. In the systemic vascular endothelium, Hbα oxidation state regulates the products and activity of colocalized eNOS at the myoendothelial junction^1^. Here, we found that increased human airway epithelial HBB gene expression is associated with increased airflow obstruction in asthmatic subjects in the SARP. We thus investigated Hb expression in human airway pseudostratified epithelium in culture. Evidence for its expression included mRNA expression (in the human airway in situ, in ALI cell culture and in ALI single-cell RNASeq analysis) and protein expression (using proteomic analysis, immunoblot, immunohistochemistry and immunofluorescence). Note that we focused primarily on cell culture preparations instead of ex vivo immunostaining to avoid Hb contamination from blood. We confirmed the presence of heme by Drabkin for the first time in airway epithelium, with the caveat that heme would be expected in several airway epithelial proteins such as cytochromes, catalase and peroxidases. Several globin proteins are expressed in the epithelium, but HBB mRNA was the most pronounced.

Theories vary regarding the roles of Hb expression in nonerythroid cells, including alveolar epithelial cells^24,25^, macrophages^33^, retinal pigment epithelial cells^27^, mesangial cells^28^, hepatocytes^29,30^, neuronal/glial cells^30^, endometrial cells^31^, and cervical cells^32^. Hemoglobin has been proposed to be an O_2_/NO sensor in stimulated macrophages^33^ and alveolar epithelial cells^24^. It has also been proposed both to facilitate oxygen transport and in scavenging oxygen and nitrogen oxides to reduce oxidative and nitrosative stress^25^. There are also proteomic and IHC data supporting for upregulation of alveolar Hb expression by androgens^34^, possibly influencing sexual dimorphism in respiratory disease. However, except in the case of the myoendothelial junction^1^, the role of somatic cell Hb remains conjectural^24^.

In lung disease, there is evidence for decreased lung epithelial Hb in patients with idiopathic pulmonary fibrosis (IPF) but not chronic obstructive pulmonary disease (COPD)^26^. Of note, decreased Hb levels in IPF have been associated with a modification of thiol group in cysteine residue (C105) of the Hbα monomer^26,35^. Hemoglobin α and Hbβ expression increased in ATII cells and MLE-15 cells after induction with hypoxia, suggesting a role in hypoxic adaptation^35^.

Here, we have identified and studied for the first time Hbs present in human airway pseudostratified epithelium. Our data suggest that it is expressed, and that it could have at least two functions. First, HBB mRNA appears to increase during maturation and ciliation of airway epithelium. On blinded analysis, HBB knockdown (maximum achieved, 50%) caused a modest decrease in ciliation, and overexpression caused complete loss of ciliation and loss of pseudostratified architecture. Though mechanisms underlying these effects may involve cellular redox chemistry, more detailed analysis will be required in the future.

Secondly, Hb may affect nitrogen oxide biology in the ciliated airway epithelium. Apical eNOS, which can signal through passive ciliary motion (airflow) as it does in other ciliated organs^23^, is co-localized with Hbβ. Air pressure and flow activate eNOS by allowing calcium entry into the cell apex, producing NO and S-nitrosothiols^3^. Here, we confirmed that eNOS can also produce NO_3_^−^ and NO_2_^−^ in the airway epithelium^22^. Specifically, upregulation of iNOS with cytomix did not dramatically increase NO_3_^−^ accumulation in the cell culture medium. CO pre-treatment almost completely ablated constitutive (eNOS-dependent)^4,22,23^ NO_3_^−^ and NO_2_^−^ accumulation in epithelial medium, consistent with its actions in the endothelium. Thus, we suspect that the paradigm demonstrated by Angelo and coworkers^36^ is relevant in the ciliated epithelium as it is at the myoendothelial junction: eNOS ultimately produces NO and S-nitrosothiols if the heme iron is oxidized, and it produces inert oxidation products if the heme iron is reduced. Though we have shown that exogenous nitrogen oxides increase cGMP accumulation in human airway smooth muscle^37^, we did not find a significant cGMP increases in our system with NOS activation (Supplementary Fig. 3), and in human, S-nitrosothiol induced airway smooth muscle relaxation is not cyclic GMP dependent^37^.

It has been shown previously that Hbβ has the cochaperone protein Hsp90, which stabilizes the immature, heme-free forms of Hbβ and then drives its heme insertion reactions in an ATP-dependent process^38^. For Hbβ, Hsp90 role is thought to be somewhat analogous to alpha hemoglobin stabilizing protein (AHSP) in the case of Hbα^39^. Here, we show that eNOS co-IP’s with Hb-β, but we do not believe that it can serve as a chaperone: it is a large, multidomain enzyme that is not routinely localized in the ER.

In vivo, the SARP data suggest that increased Hb gene expression is associated with lower lung function in humans with asthma. This could be an artifact: brush biopsies of more friable mucosa in more severe asthma could be associated with increased likelihood of identifying Hb. However, we would need to have consistently sampled erythroid progenitor cells (to identify mRNA), and these are uncommon in peripheral blood. The alternative possibility, that epithelial Hb is associated with asthmatic airflow obstruction, would suggest that the adverse effects (scavenging beneficial nitrogen oxides) predominate over beneficial effects. These possibilities will require additional studies. Note that our data may be relevant to patients with other conditions. For example, determinants of airways disease in sickle cell anemia patients (who have Hbβ mutations^40^) are poorly understood: a role for somatic cell Hb has not previously been considered.

## Conclusions

Primary human airway epithelial cells express Hb. In epithelial cells at ALI, Hbβ, colocalized with eNOS, appears to affect nitrogen oxide signaling Increased epithelial Hb gene expression is associated with increased asthmatic airflow obstruction .

## Supplementary Information

Supplementary Information.
