# Supplementary material for: Somatic cell hemoglobin modulates nitrogen oxide metabolism in the human airway epithelium
Source: Sci Rep. 2021 Jul 29;11:15498. doi: 10.1038/s41598-021-94782-5 (PMC8322277; doi:10.1038/s41598-021-94782-5)

Somatic cell hemoglobin modulates nitrogen oxide metabolism in the human airway epithelium

Nadzeya Marozkina, MD, PhD[1], Laura Smith[1], Yi Zhao, PhD[1], Joe Zein MD, PhD[4], James A. Chmiel, MD [1], Jeeho Kim, MD[3], Janna Kiselar, PhD[2], Michael D. Davis, PhD[1], Rebekah S. Cunningham, [1], Scott H. Randell, PhD[3], Benjamin Gaston, MD[1]

[1] *Herman Wells Center for Pediatric Research, Riley Hospital for Children, Indiana University School of Medicine, Indianapolis, IN,* [2]*Case Western Reserve University Cleveland, OH,* [3]*University of North Carolina School of Medicine, Chapel Hill, NC* [4]*Respiratory Institute, Cleveland Clinic, Cleveland OH.*

Supplemental material.

Supplementary Table 1 - Characteristics of SARP participants with available gene expression data stratified by Asthma status.

	**Control**	**Asthma**	**p**
n	27	128	
Age (in years)	33.01 (11.99)	37.59 (12.89)	0.092
Female Sex	15 (55.6)	85 (66.4)	0.396
Race			0.158
Caucasian	18 (66.7)	72 (60.5)	
African American	5 (18.5)	37 (31.1)	
Others	4 (14.8)	10 (8.3)	
BMI (kg/m2)	26.04 (5.31)	30.56 (7.01)	0.004
FEV_1PP	94.91 (8.44)	71.80 (22.18)	<0.001

Data are presented as n (%) for categorical variables and mean [SD] for continuous variables. Two group comparisons were made using Student's *t* test for continuous variables, and Pearson's chi-square test for categorical variables.
BMI stands for body mass index, and $FEV_1PP$for percent of predicted Forced expiratory volume in 1 sec.

Supplementary Table 2 - Multiple linear regression modeling the relationship between pre-BD FEV_1PP (dependent variable) and sex Hemoglobin gene expression in bronchial epithelial cells, adjusting for age, sex, race and body mass index.

	β-Estimate	Std. Error	p value	Model adjusted R^2	Model p value
**HBA1**				0.231	<0.0001
Log 2 (HBA1) (per unit increase in log2 HBA1)	- 21.875	7.126	0.003		
Age (in years) (per year of age)	-0.690	0.158	<0.0001		
Female Sex (vs. male sex)	6.041	4.301	0.163		
Race (AA vs. Caucasian)	-1.677	4.292	0.697		
Race (Other races vs. Caucasian)	1.747	7.084	0.247		
BMI (kg/m^2) (per unit increase in BMI)	-0.236	0.290	0.417		
**HBA2**				0.227	<0.0001
Log 2 (HBA1) (per unit increase in log2 HBA1)	-20.406	6.869	0.004		
Age (in years) (per year of age)	-0.687	0.159	<0.0001		
Female Sex (vs. male sex)	6.067	0.159	0.164		
Race (AA vs. Caucasian)	-1.682	4.307	0.697		
Race (Other races vs. Caucasian)	1.949	7.101	0.784		
BMI (kg/m^2) (per unit increase in BMI)	-0.238	0.291	0.415		
**HBB**				0.236	<0.0001
Log 2 (HBA1) (per unit increase in log2 HBA1)	-30.136	9.463	0.002		
Age (in years) (per year of age)	-0.670	0.159	<0.0001		
Female Sex (vs. male sex)	6.590	4.321	0.131		
Race (AA vs. Caucasian)	-1.939	4.286	0.652		
Race (Other races vs. Caucasian)	1.931	7.057	0.785		
BMI (kg/m^2) (per unit increase in BMI)	-0.245	0.288	0.397		
**HBD**				0.233	<0.0001
Log 2 (HBA1) (per unit increase in log2 HBA1)	-24.857	8.007	0.003		
Age (in years) (per year of age)	0.681	0.159	<0.0001		
Female Sex (vs. male sex)	6.602	4.340	0.132		
Race (AA vs. Caucasian)	-1.345	4.273	0.754		
Race (Other races vs. Caucasian)	1.918	7.074	0.787		
BMI (kg/m^2) (per unit increase in BMI)	-0.251	0.289	0.384		

HBA1 stands for Hemoglobin Subunit Alpha 1, HBA2 for Hemoglobin Subunit Alpha 2, HBB for Hemoglobin Subunit Beta, HBD for Hemoglobin Subunit Delta, BMI for body mass index, and AA for African American.

Supplementary Figure 1 – Hemoglobin gene expression in bronchial epithelial cells strongly correlate with pre-bronchodilator percent of predicted FEV_1 in asthmatics but not in controls enrolled in SARP I&II. Normalized gene expression is presented on a log 2 scale. R^2 and p value are calculated using univariate linear regression.

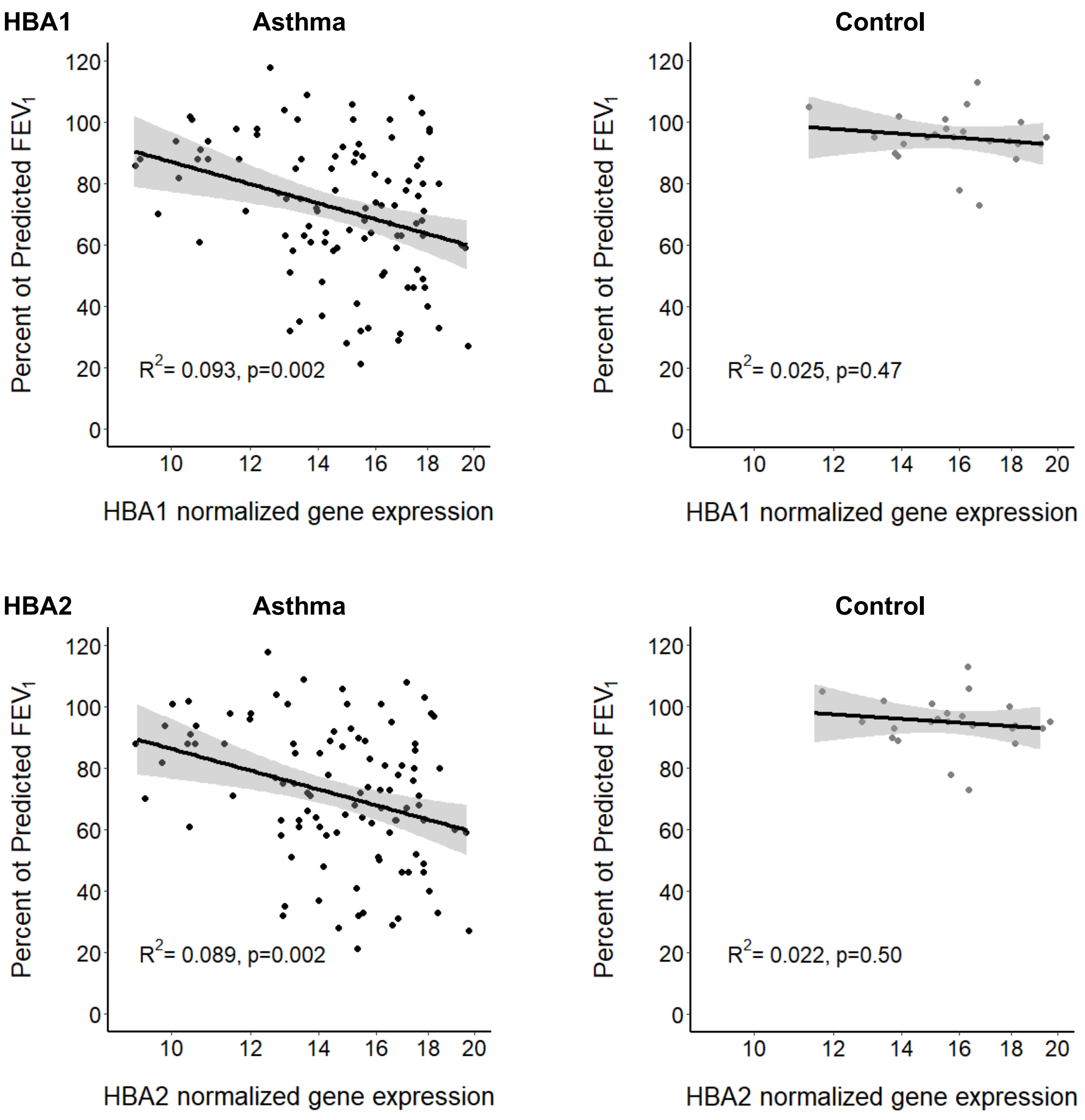

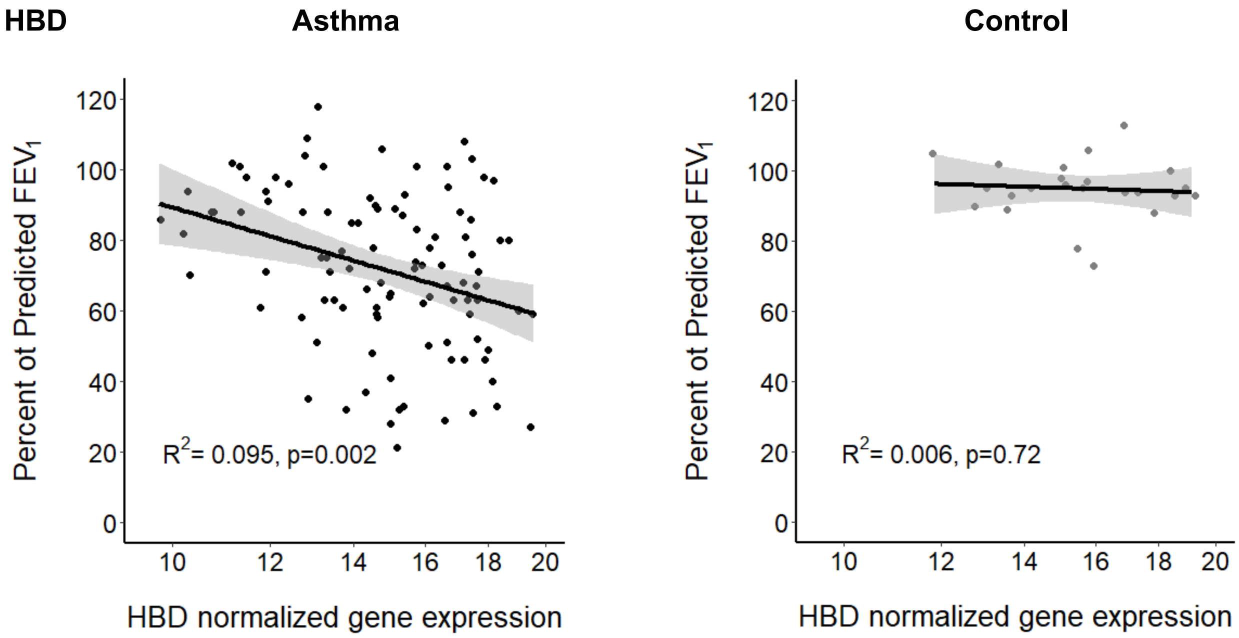

HBD
Asthma
Control
Percent of Predicted FEV1
120
100
80
60
40
20
0
R2= 0.095, p=0.002
R2= 0.006, p=0.72
10
12
14
16
18
20
HBD normalized gene expression
HBD normalized gene expression

.

Supplementary Figure 2. Uncropped blot for Figure 2.

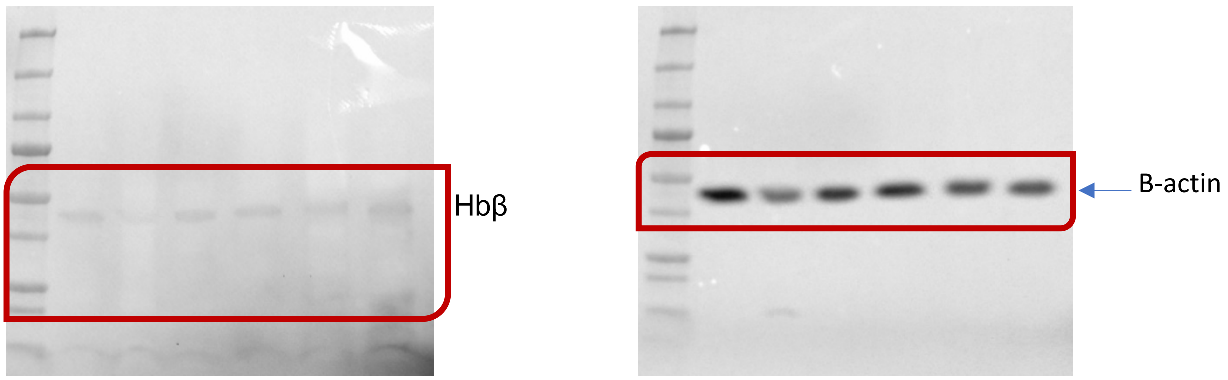

Uncropped blot for Figure 5. NHE cells were lysed and immunoprecipitated for Hbβ followed by immunoblot for eNOS. Whole cell lysate is shown.

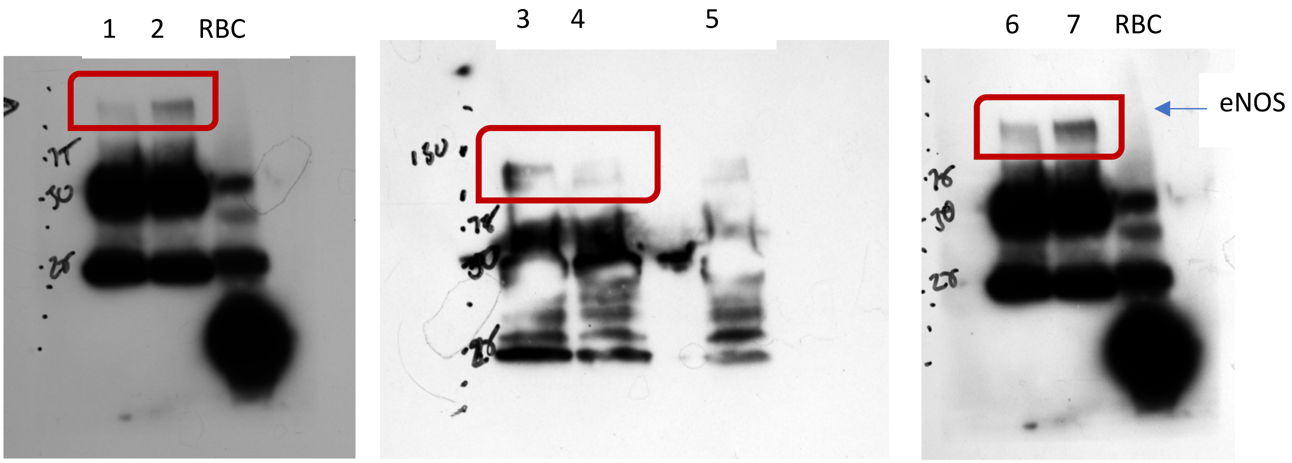

NHE cells were lysed and immunoprecipitated for eNOS followed by immunoblot for Hbβ. Whole cell lysate is shown.

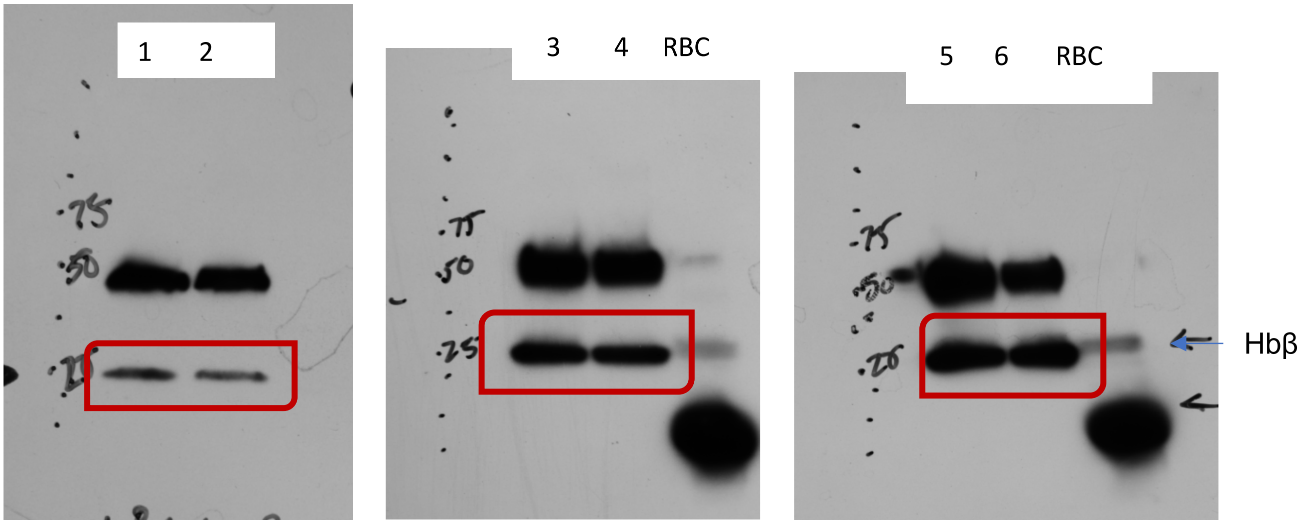

Supplementary Figure 3. cGMP levels in NHE cells treated with cytomix and CO.

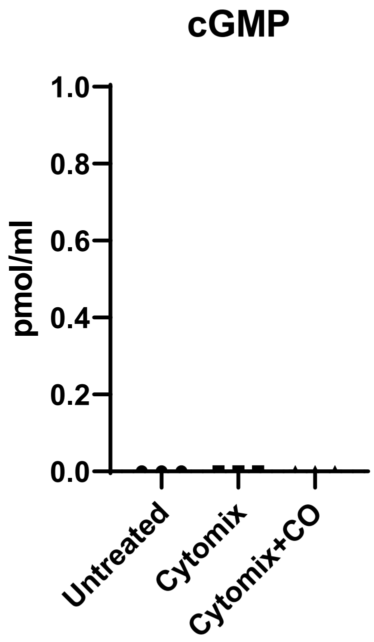

Supplement: Supplementary file 1 — Supplementary Information. [file 41598_2021_94782_MOESM1_ESM.pdf]
